# Androgen receptor may promote tumor progression via TTF-1/EGFR pathway in metastatic nasopharyngeal carcinoma

**DOI:** 10.1016/j.tranon.2026.102670

**Published:** 2026-02-02

**Authors:** Chiao-Yun Lin, Chen-Yang Huang, Kar-Wai Lui, Yin-Kai Chao, Chun-Nan Yeh, Li-Yu Lee, Yenlin Huang, Zhangung Yang, Chia-Hsun Hsieh, Hsien-Chi Fan, An-Chi Lin, Kai-Ping Chang, Chien-Yu Lin, Hung-Ming Wang, Mei Chao, Yu-Sun Chang, Hsin-Pai Li, Cheng-Lung Hsu

**Affiliations:** Chang Gung Memorial Hospital, Linkou, Taiwan

**Keywords:** Nasopharyngeal carcinoma, Epstein Barr virus, TTF-1, Patient derived xenograft, Androgen receptor

## Abstract

Nasopharyngeal carcinoma (NPC) is prevalent in Southeast Asia, including Taiwan. It exhibits higher morbidity as well as mortality in males than in females. However, the role of the androgen receptor (AR) in NPC remains unclear. In this study, AR expression was detected in most NPC cell lines and patient-derived xenografts. Treatment with enzalutamide, an antiandrogen, substantially inhibited patient-derived xenografts growth and demonstrated additive antitumor effects when combined with chemotherapy in AR-positive models. Additionally, transcriptome analysis following enzalutamide treatment revealed activation of hypoxia-inducible factor-1, steroid hormone, and AR pathways, alongside suppression of interferon and tumor necrosis factor pathways. Protein analysis further supported these transcriptomic changes. In AR-overexpressing NPC-B13 cells, AR appeared to regulate thyroid transcription factor-1 (TTF-1, encoded by *NKX2–1* gene) and its downstream target epidermal growth factor receptor (EGFR), thereby promoting cancer cell proliferation. Furthermore, chromatin immunoprecipitation suggested that AR may directly bind to the *NKX2–1* promoter to upregulate its mRNA expression. Under AR overexpression, Epstein-Barr virus (EBV) remained in a latent state, accompanied by suppression of lytic gene expression. Additionally, Epstein–Barr nuclear antigen-1 enhanced AR transactivation in a dose-dependent manner in NPC cell line reporter assays. Among 96 metastatic NPC tumor samples, AR expression was observed in 35 cases (36.5%), predominantly in males (33/83, 39.8%). AR expression correlated with poorer overall survival, with statistical significance noted in the full cohort and particularly in male patients. This study suggests that AR may promote metastatic NPC progression via the TTF-1/EGFR signaling pathway and interact with EBV to influence disease behavior, especially in males.

## Highlights

• AR expression was detected in most NPC cell lines and PDX models. Treatment with enzalutamide significantly inhibited tumor growth in PDXs.
• Enzalutamide treatment may activate the HIF-1, steroid hormone, and AR signaling pathways.
• AR appears to regulate TTF-1 and its downstream target EGFR, promoting cancer cell proliferation. AR may directly bind to the TTF-1 promoter to upregulate its mRNA expression.
• Under AR overexpression, Epstein-Barr virus (EBV) remained in a latent state.
• High AR expression was associated with poorer overall survival, especially in male patients.

## Graphical abstract

**Figure:**
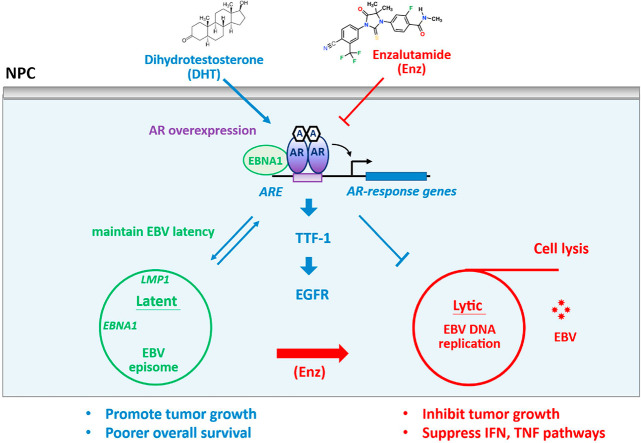

## Introduction

### Nasopharyngeal carcinoma (NPC)

NPC is a prevalent head and neck cancer in Southeast Asia [1]. Numerous etiological factors contribute to NPC pathogenesis—including individual genetics, Epstein–Barr virus (EBV) infection, exposure to carcinogens, and lifestyle characteristics [[2], [3], [4], [5]]. The expression of human leukocyte antigen genes in the major histocompatibility complex region of chromosome 6p21 is considered a primary genetic risk factor for NPC [6]. Although advancements in treatment, such as concurrent chemoradiotherapy and intensity-modulated radiotherapy, have improved the management of localized tumors, distant metastases remain a challenge in NPC management. For patients with metastatic NPC, traditional chemotherapy has been the predominant standard of care. Immune checkpoint inhibitors (ICIs)—notably, anti-programmed death-1 monoclonal antibodies—either alone or in combination with chemotherapy, have demonstrated exceptional antitumor efficacy in recurrent or metastatic NPC [7,8]. Furthermore, emerging approaches such as targeted therapy, novel immunotherapies, vaccines, cell therapy, and supportive care are promising candidates for controlling metastatic NPC [9]. Plasma EBV DNA levels are strongly correlated with clinical NPC tumor volume as well as status, and are detected in >95 % of metastatic tumors [10,11]. Additionally, other clinical parameters—including serum albumin, lactate dehydrogenase, and the number of metastatic sites (Royal Marsden Hospital score)—may stratify the clinical outcome in patients with NPC [12]. However, the limited availability of EBV-positive NPC cell lines (C666–1, NPC-43, and NPC-B13) imposes constraints on research endeavors in this domain [13,14].

### Gender differences in NPC

Global cancer statistics reveal notable gender differences in the clinical course of NPC. In particular, males experience significantly more unfavorable outcomes than females, with a 2.75-fold higher incidence and 3.25-fold higher mortality rate [15]. Females, in particular, exhibit an age-dependent survival benefit during the early and advanced stages of NPC. The highest benefit occurs at 45–60 years and declines steadily thereafter [16,17]. Despite claims suggesting that estrogen may induce EBV transcriptional activator *Bam*HI **Z E**pstein-**B**arr virus **r**eplication **a**ctivator (ZEBRA) protein expression in the lytic phase and that such activation is associated with poor NPC prognosis [18], the protective role of the estrogen-estrogen receptor pathway in females is acknowledged as a contributing factor to the observed gender differences in NPC incidence and survival [7]. Other potential reasons for why males experience poorer outcomes include older age, more advanced stage at diagnosis, more aggressive tumor biology, less optimal treatment, and greater incidence of harmful behaviors such as smoking and alcohol consumption [[19], [20], [21]]. Immune homeostasis has also been posited as a contributing factor to sex-specific differences in NPC prognosis [22]. Female patients with NPC typically respond more favorably to chemotherapy and have fewer side effects, along with different patterns of liver and kidney protein expression via sex hormone pathways [23]. Compared to them, the male patients may benefit further from ICI treatment due to high tumor mutation burden, low escape rate of immune surveillance, and less M2-tumor-associated macrophage and T-cell function associated with the estrogen-estrogen receptor pathway [24]. With regard to immune-related adverse events, however, no statistically significant difference exists between male and female patients [25].

The role of the androgen-androgen receptor (AR) pathway in these gender differences in NPC is currently unknown. In our previous studies on patient-derived xenografts (PDXs), we found that AR was differentially expressed in PDXs, especially in EBV-positive vs. EBV-negative PDXs. Furthermore, anti-androgens may inhibit the growth of PDX xenografts, and molecular mechanism analyses indicated that an interaction between AR and EBV enhances xenograft growth. These characteristics were also observed in clinical metastatic NPC patient samples, particularly in male patients with AR-positive tumors who had poor prognoses.

## Materials and methods

### Drugs

5α-dihydrotestosterone (DHT) was obtained from Sigma Chemical Co. (Saint Louis, MO, USA). Enzalutamide (ENZ) was purchased from Catalent Pharma Solutions, LLC (St. Petersburg, FL, USA). Palbociclib (Ibrance®) was obtained from Pfizer Manufacturing Deutschland GmbH (Freiburg im Breisgau, Baden-Württemberg*,* Germany). Gemcitabine was purchased from TTY Biopharma Co., Ltd. (Zhongli, Taiwan).

### Cancer cell lines and animal studies

Human cancer cell lines (C666–1 and LNCaP) were purchased from the American Type Culture Collection (Manassas, VA, USA). The NPC-B13 cell line was generated from NPC PDX-B13 in our lab [14]. EBV-positive NPC cell lines (C666–1 and NPC-B13), EBV-negative NPC cell line (HK-1), and prostate cancer cell lines (LNCaP and PC-3) were maintained in RPMI containing 10 % fetal bovine serum (FBS) and in DMEM/F-12 (Gibco, Thermo Fisher, MA, USA) supplemented with 10 % FBS, respectively. Additionally, PDX animal studies were performed as described previously [14,26]. Mice were maintained and handled in accordance with the Chang Gung Memorial Hospital (CGMH) Institutional Animal Care and Use Committee (IACUC) guidelines. All animal experiments were approved by the CGMH IACUC (IACUC No. 2022,122,019).

### Plasmid transfection and generation of NPC-B13-AROE stable cell lines

The pBabe-AR plasmid was kindly provided by Dr. Wen-Lung Ma (China Medical University/Hospital, Taiwan). Transfection was performed using the FuGENE transfection reagent (Promega, Madison, WI, USA), which was pre-equilibrated in serum-free DMEM prior to mixing with 2 μg of vector DNA. The FuGENE–DNA mixture was incubated at room temperature, then added dropwise to NPC-B13 cells. To establish stable cell lines, the culture medium was replaced 4 d post-transfection with medium containing increasing concentrations of puromycin (0, 1, 5, 10, and 15 μg/mL). Puromycin-containing medium was refreshed every 3 d for over two weeks to select for stably transfected cells, as previously described [27].

### Plasmid, reporter gene assay, and cell growth assay

NPC cell lines were used in the AR reporter assays. AR expression plasmids, PCMV-Flag-AR, and pSG5-AR, were also used. pBABE-Puro/2 plasmids were obtained from Addgene (Cambridge, MA, USA), and pBABE-Puro/2-AR was constructed according to the manufacturer’s instructions. For luciferase assays, 300 ng of MMTV-LUC reporter gene plasmid, 0.5 ng of SV40-Renilla luciferase internal control plasmid, and 500 ng pCMV-EBNA1 were transfected into NPC-B13 or HK-1 cells using a Superfect kit (Qiagen, Germantown, MD, USA). After 16 h, ethanol or 1 nm DHT was added to the wells for another 16 h. A dual luciferase reporter assay system (Promega) was used to measure the luciferase activity [28]. Furthermore, a cell growth assay ((3-(4,5-Dimethylthiazol-2-yl)-2,5-diphenyltetrazolium bromide), MTT assay) was performed as described previously [29].

### RNA interference

To generate stable *TTF-1* knockdown cell lines, NPC-B13 cell line was transfected with plasmid pLKO.1 expressing shRNA against *TTF-1* (RNAi Consortium Number [TRCN] 0000275519 and 000275520). Subsequently, the cells were selected in modified ACL-4 medium containing puromycin (2.5 μg/ml) for a total of 2 weeks. The cell lines were thereafter stably transfected, and blank lentiviral vector pLKO.1 was used as a negative control [30].

### Chromatin immunoprecipitation (ChIP) assays

A ChIP assay was performed using the CUT&RUN assay kit (#86,652, Cell Signaling Technology, Danvers, MA, USA). A total of 2 × 10^5^ NPC-B13 cells were washed and bound to concanavalin A-coated magnetic beads and permeabilized with an antibody binding buffer containing 5 % digitonin solution (#16,359, Cell Signaling Technology, Danvers, MA, USA). The cells were subsequently incubated with 0.5 μg of AR antibody C-19 (Santa Cruz, Santa Cruz, CA, USA) overnight at 4 °C. After completion of the antibody reaction, the cells were washed twice with digitonin buffer containing 5 % digitonin solution and incubated with protein AG–micrococcal nuclease (MNase) for 1 h at 4 °C. Thereafter, the cells were washed again with digitonin buffer, and protein AG–MNase digestion was initiated by adding 2 mM CaCl_2_, following which the samples were incubated on ice for 30 min. Subsequently, the reaction was terminated by adding 1 × stop buffer containing 5 % digitonin solution, ribonuclease A (50 μg/ml), and Spike-In DNA (5 pg). The CUT&RUN fragments were released by incubation at 37 °C for 10 min, followed by centrifugation at 4 °C and 16,000 *g* for 2 min. Thereafter, the supernatant was collected, and DNA was purified using DNA purification buffers and spin columns (#14,209, Cell Signaling Technology, Danvers, MA, USA). DNA quantification was performed via PCR (KAPA HiFi) and analysis on a 1.2 % agarose gel.

### Patient samples

Clinical metastatic samples from 96 patients with NPC were retrospectively collected between January 2007 and February 2024 in CGMH. This study was approved by the Institutional Review Board of CGMH (CGMH IRB No. 202101677B0).

### PDX drug screening

The protocols for drug sensitivity tests have been previously described [26]. Briefly, NPC PDXs were implanted subcutaneously in the flank region of anesthetized NOD/SCID mice. Once the tumors reached a diameter of approximately 1 cm, treatment with various candidate chemicals was initiated simultaneously. Tumor dimensions were measured twice a week using calipers, and tumor volume was calculated using the formula: tumor volume (mm³) = tumor length (mm) × [tumor width (mm)]² × 0.5. Tumors were then harvested for further analysis. Four to six mice per group (with or without chemical treatment) were used, and the mice were sacrificed 4–6 weeks after chemical injection.

### Bulk RNA sequencing and differential expression gene analysis

RNA was extracted from the cells as described previously [31]. The raw fastq reads were aligned to a human–mouse–EBV hybrid reference genome by using the STAR software (GRCh38, GRCm39, and EBV-GD1 build) [32]. Furthermore, alignment quality was evaluated using Qualimap 2 [33]. The reads aligned to the mouse genome were removed prior to downstream analysis. Transcript abundance was evaluated using the STAR software with the default parameters. Additionally, the differentially expressed gene (DEG) analysis and gene ontology enrichment were performed as described previously [31]. The EBV GD1 strain (GenBank accession no. AY961628) was chosen as the reference genome for sequence alignment and quantification [34]. Gene counts were later converted to Reads Per Kilobase per Million mapped reads (RPKM) for DEG analysis.

### Antibodies

The following antibodies were used in this study: AR, C-19; γ Enolase, sc-21,738 (Santa Cruz Biotechnology, Santa Cruz, CA, USA); GLUT1, 07–1401 (Merck Millipore, Daejeon, South Korea); c-Fos, AF5354; IFIT3, DF8963 (Affinity Biosciences, Cincinnati, OH, USA); GAPDH, GTX100118 (Genetex, Hsinchu, Taiwan); TTF-1, ab76013; EGFR, ab52894; and pEGFR, ab40815 (Abcam, Cambridge, UK).

### Statistical analysis

The median survival time of patients with NPC was estimated as the first observed event (death) at which the Kaplan–Meier survival probability was equal to 1.5. Furthermore, overall survival was calculated as the period from recurrence or metastasis to death, estimated by Kaplan–Meier plotting, and compared via log-rank test. Two-tailed p-values below 0.05 were considered statistically significant.

## Results

### AR expression in NPC PDX and cell lines

**Fig. 1:**
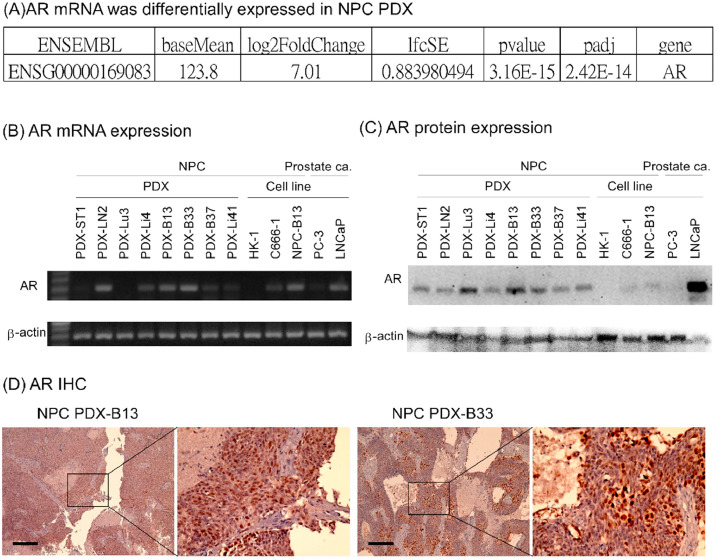
**AR expression in NPC cell lines and PDXs.** (A) AR mRNA was differentially expressed in EBV-positive NPC PDX-B13 compared to EBV-negative NPC PDX-Li41. (B) AR mRNA expression in PDXs and cell lines. (C) AR protein expression in PDXs and cell lines. (D) AR immunohistochemistry (IHC) stains in NPC PDXs.

Our previous NPC PDX-related studies found that AR mRNA had high expression levels with a 130-fold difference (2^7.01^) in NPC PDX-B13 (EBV-positive) vs. NPC PDX-Li41 (EBV-negative) (Fig. 1A). The present study elucidated AR expression levels in other available NPC cell lines and PDXs. Notably, AR mRNA and protein expression levels were almost universally detected in the NPC cell lines and PDXs that were tested (Fig. 1B and 1C). AR expression was also observed in NPC PDX tumor via immunohistochemical staining (Fig. 1D). These data indicated that AR is broadly expressed in commonly used NPC cell lines.

### Anti-androgen effects in AR-positive NPC cancer cells

**Fig. 2:**
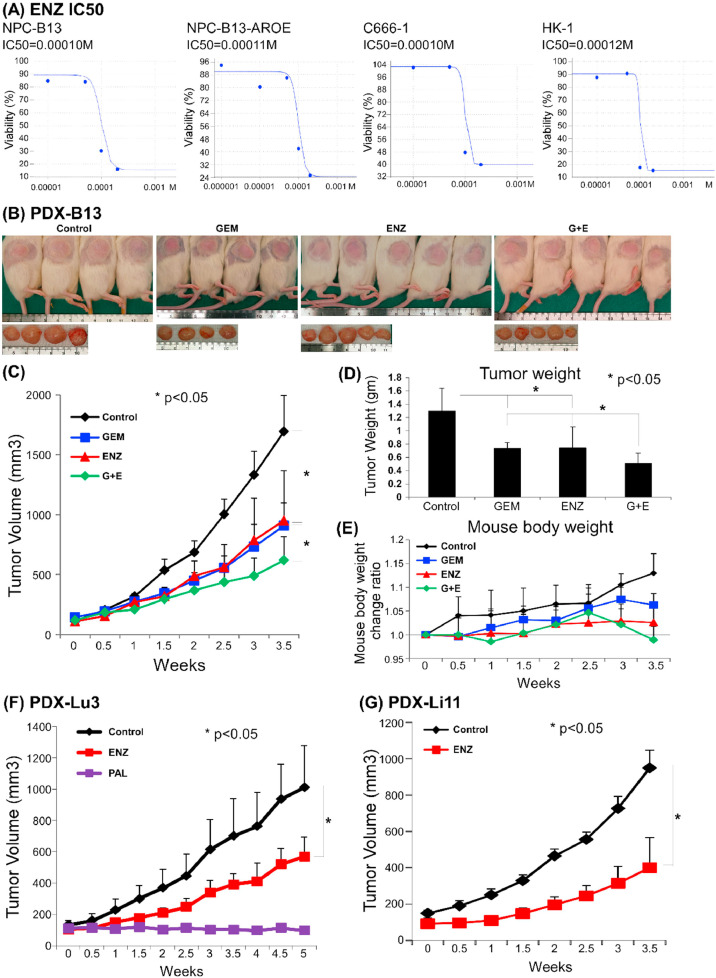
**NPC cell lines and PDXs drug test.** (A) ENZ IC_50_ in four NPC cell lines. (B∼E) PDX-B13. (B) PDX-B13 gross tumor size (C) PDX-B13 tumor volume (D) PDX-B13 tumor weight (E) PDX-B13 mouse body weight (F) PDX-Lu3 tumor volume (G) PDX-Li11 tumor volume. Abbreviations: GEM, gemcitabine; ENZ, enzalutamide; PAL, palbociclib.

ENZ is a potent anti-androgen used clinically for treating prostate cancer. IC_50_ for the NPC cell lines used in this study was observed to be approximately 0.0001 M (Fig. 2A). Furthermore, the study tested the antitumor effects of ENZ in NPC PDX-B13, which expresses AR at high levels. It was found that ENZ significantly inhibited the tumor growth compared to the control (Fig. 2B–D). Moreover, treatment with ENZ and gemcitabine had an additive antitumor effect. ENZ also significantly inhibited tumor growth in the other AR-positive PDXs (PDX-Lu3 and PDX-Li11) used in this study (Fig. 2F and 2G). Consequently, these data suggested that ENZ could inhibit tumor growth in AR-positive NPC PDX.

**Fig. 3:**
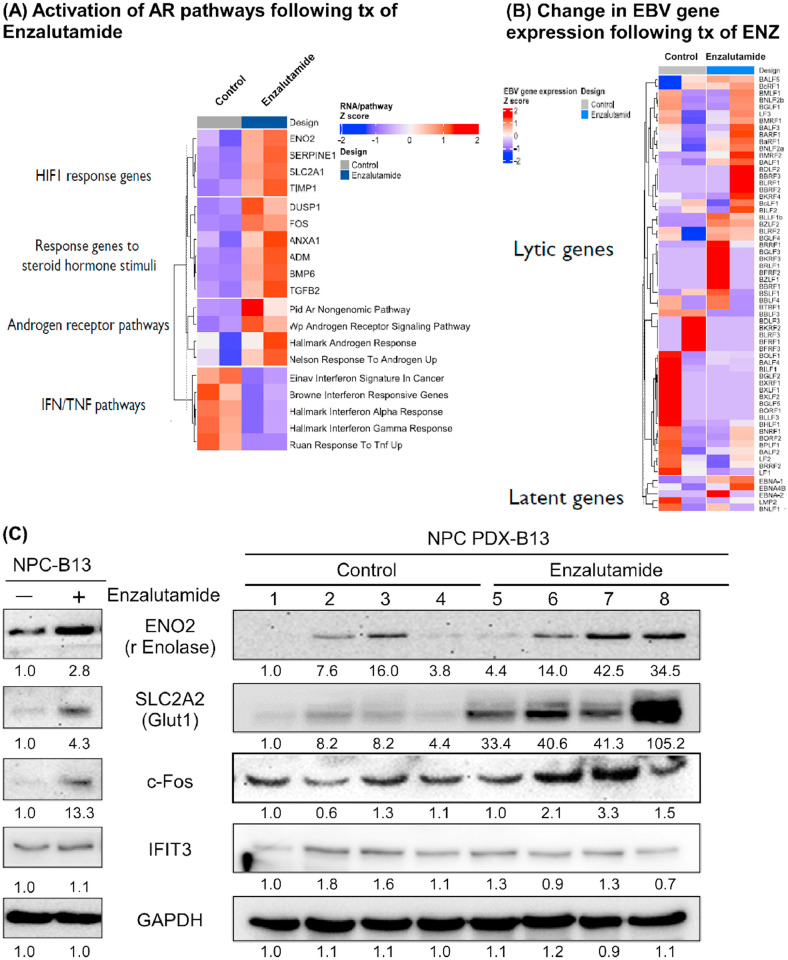
**Gene and protein expression in NPC PDX-B13 following ENZ treatment.** (A) ENZ-treated PDX tumor cells showed activation of the HIF1 signaling pathway, steroid hormone response, and androgen receptor (AR) pathway, along with suppression of interferon (IFN) and tumor necrosis factor (TNF) signaling pathways. (B) EBV RNA expression profiling post-treatment revealed a mixed pattern of changes in both latent and lytic phase gene expression. (C) Protein expression of selected candidate markers was altered following ENZ treatment in both NPC-B13 cells and NPC PDX-B13 tumors.

After exposing PDX-B13 to ENZ, transcriptome analysis revealed that hypoxia-inducible factor (HIF) 1, steroid hormone stimuli, and AR pathways were activated, whereas the IFN/TNF pathways were suppressed (Fig. 3A). In PDX-B13, ENZ treatment activated EBV latent phase genes, such as Epstein-Barr Nuclear Antigen 1 (*EBNA1*) and *EBNA4B*, but EBV lytic phase gene responses were heterogeneous (Fig. 3B).

Furthermore, the NPC-B13 cell line was observed to be AR- and EBV-positive. ENZ treatment increased the expression of HIF1-responsive genes coding proteins, such as enolase 2 (coded by *ENO2*) and GLUT1 (coded by *SLC2A1*), as well as proteins coded by steroid hormone stimulus-responsive genes, such as c-Fos (coded by *FOS*). However, there was no change in the IFN/TNF pathway-related protein IFIT3 (Fig. 3C, left panel). Similar responses were also observed in NPC PDX-B13 (Fig. 3C, right panel). Therefore, these results demonstrated that protein expression correlates with mRNA expression responses to ENZ treatment.

### AR participation in TTF-1/EGFR pathway to enhance NPC cell growth

**Fig. 4:**
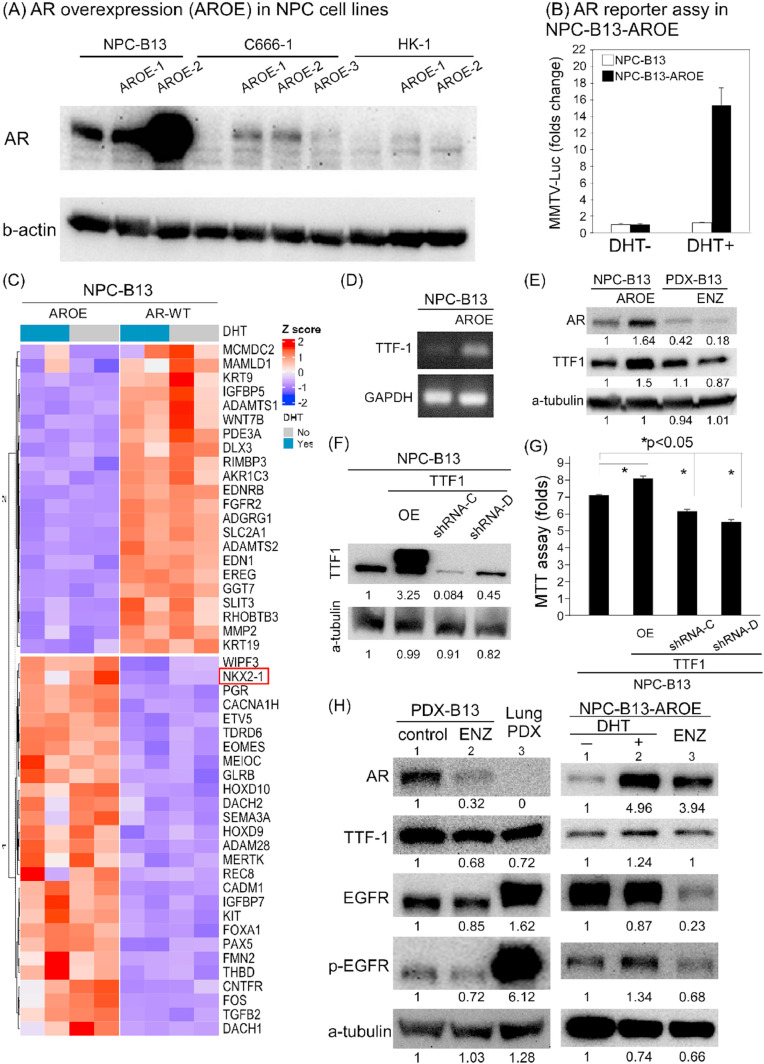
**AR may go through TTF-1/EGFR pathway to enhance NPC cell growth.** (A, B) In AR overexpression NPC cells, NPC-B13-AROE, AR transcription activity may be enhanced in reporter assay. (C) Transcriptome analysis revealed *NKX2–1*(*TTF-1*) up-regulated in NPC-B12-AROE cells. (D) *TTF-1* expression correlated with AR in mRNA level in NPC-B13-AROE. (E) TTF-1 expression correlated with AR in protein level in NPC-B13-AROE and PDX-B13 after ENZ treatment. (F) TTF-1 protein expression may be reduced by *TTF-1*-shRNA. (G) TTF-1 expression may correlate with cancer cell growth in NPC-B13. (H) AR may regulated TTF-1/EGFR protein expression in NPC PDX-B13 and NPC-B13-AROE cells.

Although AR is ubiquitously expressed in NPC cell lines, its transcriptional activity—as measured by a reporter assay—is not activated by DHT in NPC-B13 cells. To investigate AR function, a stable NPC-B13 cell line with constitutive AR overexpression (NPC-B13-AROE) was generated by transfecting pBabe-AR and selecting with puromycin. Successful overexpression was confirmed via both protein expression and AR transcriptional activity using a reporter assay (Fig. 4A and 4B).

Moreover, comparative transcriptomic analysis between parental NPC-B13 and NPC-B13-AROE cells revealed differential mRNA expression profiles. In NPC-B13-AROE, pathways related to neurotransmission, chemokines, and amino acid metabolism/transport were upregulated, whereas glucose and lipid metabolism pathways were downregulated (Supplementary Fig. 1). Among the upregulated genes, *NKX2–1* (also known as thyroid transcription factor-1 or TTF-1) was identified as a candidate AR-regulated gene (Fig. 4C). TTF-1 is a transcription factor essential for lung, thyroid, and ventral forebrain development [35], and is known to be highly expressed in non-squamous lung cancers [36].

A correlation between AR and *TTF-1* mRNA expression was observed via semi-quantitative PCR (Fig. 4D). This was also reflected at the protein level in NPC-B13-AROE and PDX-B13 models treated with ENZ (Fig. 4E). Additionally, RNA interference-mediated knockdown of *TTF-1* in NPC-B13 cells confirmed a functional role of TTF-1 in promoting cancer cell proliferation (Fig. 4F and Fig. 4G). Previous studies have identified EGFR (epithelial growth factor receptor) as a downstream target of TTF-1 [37]. In the present study, the expression of TTF-1 and phosphorylated EGFR (p-EGFR) was found to be correlated with AR expression in PDX-B13, following ENZ treatment. A similar relationship was also observed in NPC-B13-AROE cells (Fig. 4H).

### AR can directly regulate *TTF-1* mRNA expression

**Fig. 5:**
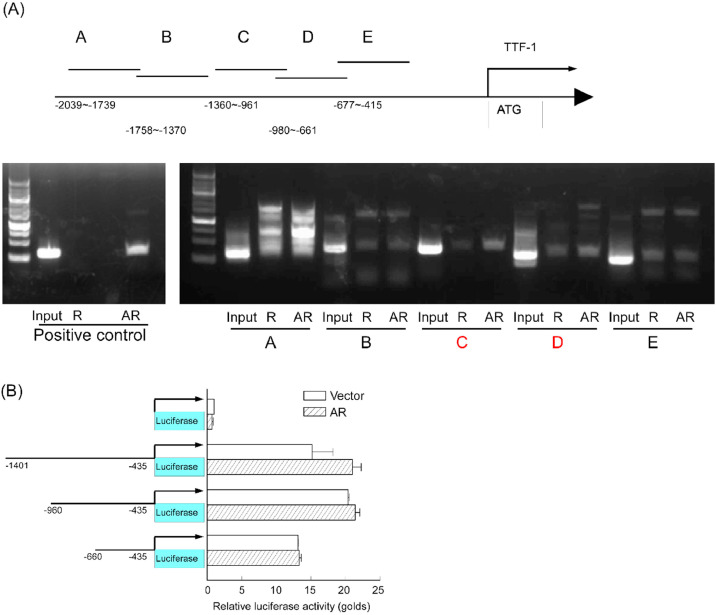
**AR can directly regulate *TTF-1* mRNA expression.** (A) DHT Induces the Recruitment of AR preferentially to the *TTF-1* promoter. diagram illustrating the 1.7-kb upstream regulatory region of the *TTF-1* gene, with regulatory elements indicated above and the various fragments amplified in the ChIP assay (labeled A to E). The amplification efficiency of the primer pairs for these fragments was assessed by PCR under identical cycling conditions. NPC-B13 cells were treated with 1 μM DHT for 24 h, and ChIP assays were performed using the indicated antibodies (R: rabbit IgG, AR: AR antibody) to evaluate AR chromatin occupancy across the 1.7-kb *TTF-1* sequence. The *PSA* promoter was used as a positive control to confirm AR binding to its target site. Arrows indicate the PCR product. (B) Delete the candidate AR binding site may influence *TTF-1* transcription activity.

Given that both AR and TTF-1 are transcription factors, and that AR expression correlates with TTF-1 protein levels, we hypothesized that AR may directly regulate *TTF-1* transcription. ChIP assays demonstrated that AR bound to the *TTF-1* promoter region. Moreover, deletion of putative AR binding sites reduced *TTF-1* mRNA expression in reporter assays (Fig. 5A and 5B). Collectively, these findings suggest that AR may enhance metastatic NPC tumor growth via the TTF-1/EGFR signaling pathway.

### Interplay between AR-EBNA1

**Fig. 6:**
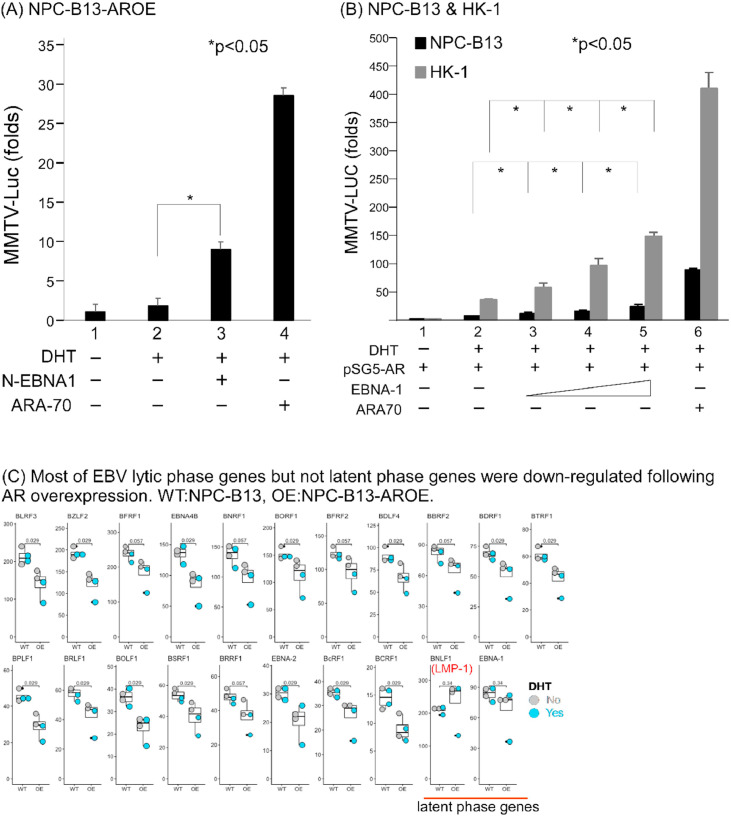
**EBNA1 can enhance AR transcriptional activity and AR overexpression may keep EBV in the latent phase.** (A) Reporter assay of NPC-B13-AROE (B) Reporter assays of NPC-B13 and HK-1. ARA-70 served as a positive control. (C) AR overexpression may keep EBV in the latent phase and from lytic phase gene expression. Abbreviation: DHT, dihydrotestosterone.

Oncoviruses may interact with transcriptional factors to enhance tumor progression [38]. This study investigated whether this phenomenon occurs between EBV and AR in NPC. Exposure to DHT elicited EBNA1 activation of AR transcription in NPC-B13-AROE. DHT increased AR transcriptional activity in a dose-dependent manner in NPC-B13 and HK-1 NPC cell lines (Fig. 6A and 6B). Furthermore, NPC-B13-AROE stably expressed EBV latent phase genes—such as *EBNA-1* and *LMP-1*—and exhibited a decrease in lytic gene expression (Fig. 6C). These data suggested that AR may maintain EBV in a latent phase and suppress a shift to the lytic phase, which would kill the host cancer cells. Moreover, EBV may interact with AR in EBV-positive and AR-positive NPC and potentially enhance tumor progression.

### AR expression correlated with poor prognosis in male metastatic NPC patients

**Fig. 7:**
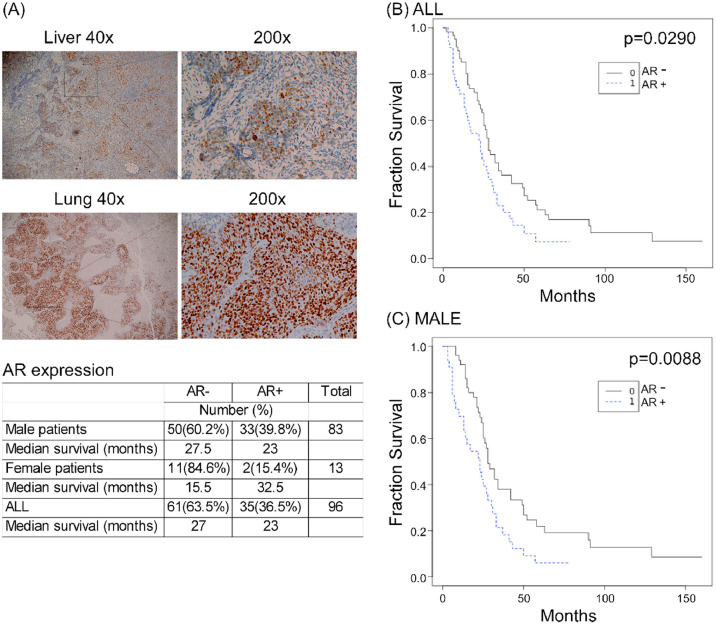
**AR was a prognostic factor in metastatic NPC patients.** (A) IHC staining of AR in patients with metastatic NPCs to the liver (upper) and lung (lower). AR expression in metastatic NPC tumors and was more common in male. AR expression in metastatic NPC patients was associated with a poor prognosis in all patients (B) and male patients (C).

NPC is known to be more prevalent in males and is associated with a poor prognosis. The present study investigated the correlation between AR expression in metastatic NPC patients and overall survival. AR expression was examined via immunohistochemical staining of metastatic NPC tumors obtained from the CGMH tumor bank in Linkou, Taiwan. It was observed that the AR expression was high in some metastatic tumors from the lung and liver (Fig. 7A). Ninety-six metastatic tumor tissues were assayed for AR expression, among which 35 (36.5 %) were positive for the same and significantly associated with shorter median survival time (AR-positive: 23 months; AR-negative: 27 months, *p* = 0.0290) (Fig. 7B). When tumors solely from male patients (*n* = 83) were analyzed, the incidence of AR expression was higher (33/83, 39.3 %) and these patients with AR-positive tumors were more significantly associated with poor overall survival than the patients with AR-negative tumors (*p* = 0.0088). Overall, these data indicated that AR expression is a predictive factor for poor prognosis in patients with metastatic NPC, especially male patients.

## Discussion

### Androgen-AR pathway may disadvantage metastatic NPC patients, particularly males

Gender disparities with male predominance have been reported for several types of solid tumors, except those of the sex organs, thyroid, and gall bladder. Higher incidences in males may be explained by gender-specific behaviors, anthropometrics, lifestyles, and medical histories [39]. Males with cancer also have shorter survival times than females across most cancer types [40]. AR mRNA expression has been reported in certain clinical NPC tumors, wherein AR may induce long non-coding RNA (lncRNA) LINC01503, thereby facilitating NPC proliferation and metastasis via the SFPQ-FOSL1 pathway [41]. Furthermore, kinesin-like protein 23 (KIF23) is directly regulated by AR, whereby it activates the downstream Wnt/β-catenin pathway and enhances NPC tumor progression [42]. The current study demonstrated that AR was highly expressed in NPC *ex vivo* models, including PDXs and cell lines. In AR-expressing PDXs, antiandrogen inhibited xenograft growth and activated HIF as well as steroid hormone pathways. Moreover, antiandrogen treatment exhibited an additive antitumor effect when combined with chemotherapy. These results provide new evidence that gender disparities in the prevalence and survival of NPC may arise from the protective effects of estrogen-ER pathway in females and the disadvantage of androgen-AR pathway activation in males with NPC.

### AR may enhance metastatic NPC growth through regulation of the TTF-1/EGFR pathway

TTF-1 is a transcription factor that serves as both a diagnostic and prognostic marker in non-squamous cell lung cancer [36]. In lung adenocarcinoma, TTF-1 has been shown to promote tumor growth via activation of the EGFR pathway [37]. In prostate cancer, certain tumor cells can undergo neuroendocrine differentiation, co-expressing AR and the neuroendocrine marker TTF-1, although the interaction between these two proteins has not been well characterized [43]. The present study demonstrated that AR may promote metastatic NPC growth through the TTF-1/EGFR signaling axis. Manipulation of AR levels influenced both TTF-1 and EGFR protein expression and was correlated with enhanced cancer cell proliferation. Furthermore, ChIP assays revealed that AR may directly bind to the promoter region of *TTF-1*, thereby regulating its mRNA expression. These findings provided mechanistic insight into how AR expression contributes to tumor progression and may explain its association with poor prognosis in patients with metastatic NPC.

### ENZ treatment may activate the HIF1 and steroid hormone pathways in AR±EBV± NPC

Tumor hypoxia may activate the HIF1 pathway and increase the expression of all glycolytic enzymes, including ENO2 and glucose transporters GLUT1 (encoded by SLC2A1) [44]. ENO2 is a crucial glycolytic enzyme involved in cancer metabolic processes, whereas HIF1 is a transcription factor directly involved in glycolysis. HIF1 binds to sequences at positions −154 to −143 and −131 to −116 within the ENO2 promoter region [45]. The present study found that ENZ increased ENO2 and GLUT1 expression by blocking the AR signaling pathway. The interplay between hypoxia and AR may alter cellular metabolism and confer resistance to androgen or AR-targeted therapies in prostate cancer [46]. Furthermore, androgen may suppress c-Fos expression by repressing the PKC/MEK/ERK/ELK-1 pathway in prostate cancer [47]. This study established that antiandrogen treatment increases c-Fos mRNA and protein in AR+ NPC. Overall, these results explain the metabolic and signaling changes in AR+ NPC tumor in response to ENZ treatment.

### Interplay between AR and EBV enhances NPC tumor progression

Oncoviruses may interact with the androgen-AR pathway to increase tumor growth and contribute to associations between gender and outcomes in liver cancer animal models [38]. EBNA1 regulates the EBV genome during DNA replication, mitotic segregation, and EBV transcriptional activity [48]. EBNA1 is the only EBV protein expressed in all EBV-positive tumors and during the latent phase in proliferating cells. Oftentimes, it is the only detectably expressed EBV protein, although it may also be expressed during the lytic phase of EBV [49]. EBNA1 directly or indirectly regulates host genes involved in translation, transcription, and cell signaling pathways—including the STAT1 and TGFβ [50], AP-1 [51], NF-κB [52]—as well as BMP-related pathways [[53], [54], [55]]. EBNA1-targeting therapy has demonstrated potential benefit in EBV-related cancers [56,57]. The current study found that EBNA1 increased AR transcription activity, whereas AR helped maintain EBV in the latent phase in AR+ EBV+ NPC tumors. An effect of these two pivotal factors may be tumor progression leading to poor prognosis in patients with metastatic NPC tumors that are AR-positive and EBV-positive, especially in males. These findings underscore the complex interplay between hormonal pathways and molecular mechanisms that affect NPC outcomes, thus providing valuable insights for future research and potential therapeutic interventions.

Although AR expression may influence outcomes in male patients with metastatic cancer and could serve as a potential therapeutic target, this finding is limited by the small number of cases analyzed and the low tissue proof ratio among patients with metastatic NPC. In female metastatic NPC patients, the role of AR was unknown. Moreover, the role of estrogen receptor expression in female metastatic NPC patients remains unclear. These hypotheses may be further validated through more comprehensive studies with a larger number of tissue samples, clinical correlation, and research material establishment to clearly elucidate the relationship between sex hormone receptors-EBV interaction and gender-related differences associated with cancer.

## Funding

This work was supported by grants from the Chang Gung Memorial Hospital, Taiwan (CMRPG3M0231∼3, C.-L. Hsu); CMRPD1L0351 and CMRPD1N0161 for H.-P. Li; National Science and Technology Council, Taiwan (NSTC-113–2314-B-182–019 for C.-L. Hsu; NSTC-112–2320-B-182–041-MY3 for H.-P. Li).

## Ethics declarations

This study was approved by the Institutional Review Board of CGMH (CGMH IRB No.201900135A3). Mice were maintained and handled in accordance with the Chang Gung Memorial Hospital (CGMH) Institutional Animal Care & Use Committee (IACUC) guidelines. Animal experiments were approved by the CGMH IACUC.

## Consent for publication

Not applicable.

## CRediT authorship contribution statement

**Chiao-Yun Lin:** Investigation, Formal analysis, Data curation. **Chen-Yang Huang:** Methodology, Formal analysis. **Kar-Wai Lui:** Resources. **Yin-Kai Chao:** Resources. **Chun-Nan Yeh:** Resources. **Li-Yu Lee:** Formal analysis. **Yenlin Huang:** Formal analysis. **Zhangung Yang:** Resources. **Chia-Hsun Hsieh:** Resources. **Hsien-Chi Fan:** Formal analysis. **An-Chi Lin:** Formal analysis. **Kai-Ping Chang:** Resources. **Chien-Yu Lin:** Resources. **Hung-Ming Wang:** Resources. **Mei Chao:** Resources, Methodology. **Yu-Sun Chang:** Supervision. **Hsin-Pai Li:** Writing – original draft, Methodology, Funding acquisition, Conceptualization. **Cheng-Lung Hsu:** Writing – review & editing, Methodology, Funding acquisition, Data curation, Conceptualization.

## Declaration of competing interest

The authors declare the following financial interests/personal relationships which may be considered as potential competing interests: Chang Gung Memorial Hospital, Taiwan; the Ministry of Science and Technology, Taiwan; Ministry of Education, Taiwan

## Appendix Supplementary materials

Image, application 1
