# Supplementary material for: Androgen receptor may promote tumor progression via TTF-1/EGFR pathway in metastatic nasopharyngeal carcinoma
Source: Transl Oncol. 2026 Feb 2;65:102670. doi: 10.1016/j.tranon.2026.102670 (PMC12887719; doi:10.1016/j.tranon.2026.102670)
Supplement: Supplementary file 1 [file mmc1.pdf]

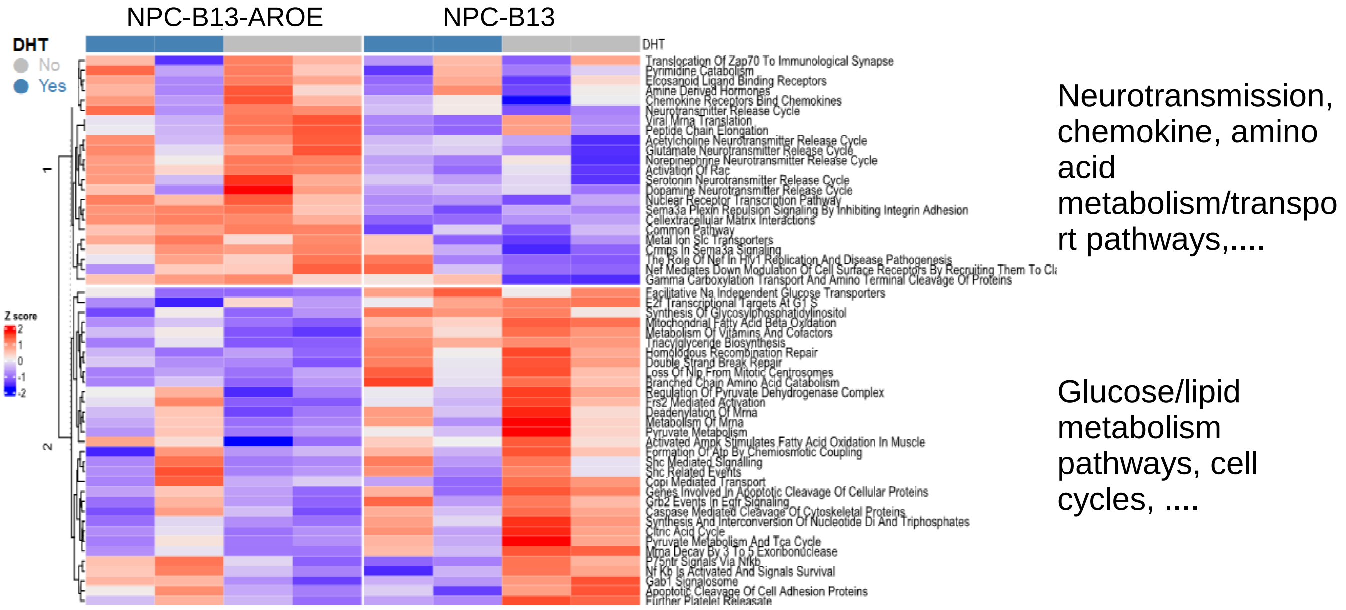

Supplement Fig. 1 Genes expression change following AR overexpression. Pathways related to neurotransmission, chemokines, and amino acid metabolism/transport were upregulated, while glucose and lipid metabolism pathways were downregulated.
